# Microbial Nitrogen Transformation Potential in Sediments of Two Contrasting Lakes Is Spatially Structured but Seasonally Stable

**DOI:** 10.1128/msphere.01013-21

**Published:** 2022-02-02

**Authors:** Kathrin B. L. Baumann, Raoul Thoma, Cameron M. Callbeck, Robert Niederdorfer, Carsten J. Schubert, Beat Müller, Mark A. Lever, Helmut Bürgmann

**Affiliations:** a Department of Surface Waters-Research and Management, Eawag, Swiss Federal Institute for Aquatic Science and Technology, Kastanienbaum, Switzerland; b Institute of Biogeochemistry and Pollutant Dynamics (IBP), ETH Zurich, Zurich, Switzerland; University of Wisconsin-Madison

**Keywords:** metagenomics, microbial ecology, freshwater, pore water, DNRA, nitrification, denitrification, anammox, comammox, nitrogen transformation

## Abstract

The nitrogen (N) cycle is of global importance, as N is an essential element and a limiting nutrient in terrestrial and aquatic ecosystems. Excessive anthropogenic N fertilizer usage threatens sensitive downstream aquatic ecosystems. Although freshwater lake sediments remove N through various microbially mediated processes, few studies have investigated the microbial communities involved. In an integrated biogeochemical and microbiological study on a eutrophic and oligotrophic lake, we estimated N removal rates from pore water concentration gradients in sediments. Simultaneously, the abundance of different microbial N transformation genes was investigated using metagenomics on a seasonal and spatial scale. We observed that contrasting nutrient concentrations in sediments were associated with distinct microbial community compositions and significant differences in abundances of various N transformation genes. For both characteristics, we observed a more pronounced spatial than seasonal variability within each lake. The eutrophic Lake Baldegg showed a higher denitrification potential with higher *nosZ* gene (N_2_O reductase) abundances and higher *nirS*:*nirK* (nitrite reductase) ratios, indicating a greater capacity for complete denitrification. Correspondingly, this lake had a higher N removal efficiency. The oligotrophic Lake Sarnen, in contrast, had a higher potential for nitrification. Specifically, it harbored a high abundance of *Nitrospira*, including some with the potential for comammox. Our results demonstrate that knowledge of the genomic N transformation potential is important for interpreting N process rates and understanding how the lacustrine sedimentary N cycle responds to variations in trophic conditions.

**IMPORTANCE** Anthropogenic nitrogen (N) inputs can lead to eutrophication in surface waters, especially in N-limited coastal ecosystems. Lakes effectively remove reactive N by transforming it to N_2_ through microbial denitrification or anammox. The rates and distributions of these microbial processes are affected by factors such as the amount and quality of settling organic material and nitrate concentrations. However, the microbial communities mediating these N transformation processes in freshwater lake sediments remain largely unknown. We provide the first seasonally and spatially resolved metagenomic analysis of the N cycle in sediments of two lakes with different trophic states. We show that lakes with different trophic states select for distinct communities of N-cycling microorganisms with contrasting functional potentials for N transformation.

## INTRODUCTION

Nitrogen (N) is an essential nutrient, and microbes play central roles in the environmental N cycle. In most environments on earth, N-fixing microbes dominate the conversion of dinitrogen (N_2_) to reactive N (N compounds readily available for biological conversion; e.g., NO_3_^−^, NO_2_^−^, NH_4_^+^, and N_2_O). The different reactive N compounds are used as electron acceptors or donors in several microbial metabolic pathways. In addition, the availability of the essential nutrient N limits rates of primary production in many terrestrial and aquatic ecosystems, including coastal oceans (1–3). As a result, excessive anthropogenic reactive N input, e.g., from runoff of agricultural fertilizer, human wastewater, and fossil fuel combustion, increases biomass production and organic carbon loads in aquatic ecosystems and can lead to the occurrence of harmful algal blooms and anoxia (4, 5). Despite its small area, Switzerland is an essential headwater system of European rivers and a nonnegligible N source. N loads from atmospheric deposition (44 kt year^−1^), mineral fertilizers (52 kt year^−1^), and sewage (43 kt year^−1^) are about equally responsible for the N contamination of ecosystems in Switzerland (6). Atmospheric deposition rates on the Swiss Plateau, locally exceeding 40 kg of N ha^−1^ year^−1^, are among the highest in the world (6, 7). Today, Switzerland exports 63 kt year^−1^ of dissolved N via the Rivers Rhine and Rhone (an average from 1995 to 2013) (8). This load corresponds to 1.3% of the total N exports of Europe to the seas (4,761 kt year^−1^) (9).

Lakes have been identified as important N sinks that convert up to 90% of reactive N to less bioavailable N_2_ gas and, thus, substantially reduce N loads to oceans (10). N removal occurs through several microbially mediated processes, mainly within the oxic-anoxic transition zone. This zone extends across the first few millimeters of sediments in most lakes. The main N loss processes include denitrification and anaerobic ammonia oxidation (anammox), both of which produce N_2_ as an end product, as well as long-term burial of N-containing organic matter (sequestration) (11–17). Furthermore, the effectiveness of reactive N conversion to N_2_ depends on interactions with other N-cycling reactions that produce or compete for the same substrates. However, a thorough understanding of the environmental controls of these N removal processes is lacking despite being essential to understanding and predicting N removal by lakes, both today and under conditions of global change in the future.

Many different environmental drivers influence the reactive N transformation processes in lakes. These include NO_3_^−^ concentrations, organic matter (OM) input, remineralization rates, and availability of electron acceptors besides NO_3_^−^ [e.g., O_2_, SO_4_^2−^, Fe(III), Mn(IV), and CO_2_] (1, 4, 10, 11, 16, 18–23). Most of these factors are intricately linked to the overall trophic status of lakes. For example, Finlay et al. (12) reported 7-fold higher N removal rates in eutrophic than in oligotrophic lakes. Others found that interlake variations in denitrification rates were positively correlated with water residence times and N loads (5, 24, 25).

There is a considerable lack of understanding about the controls on microbial communities involved in N transformation processes in lake sediments. Microbial ecology has made substantial progress in recent decades in identifying, characterizing, and quantifying key genes of microbial N-cycling enzymes (N transformation genes) in environmental systems using various molecular tools (15, 17, 26–29). This has improved understanding of the environmental controls of nitrification, complete ammonia oxidation (comammox), assimilatory and dissimilatory nitrate reduction to ammonia (ANRA and DNRA), denitrification, anammox, and N_2_ fixation (1, 14, 22, 27, 28, 30, 31). Metagenome sequencing allows for a more complete view of microbial N cycling by enabling the study of multiple N gene families and other pathways in environmental samples in parallel (32). Fierer et al. (33), for example, noted a significant change in the microbial community and its metabolic function (e.g., increase of DNA/RNA replication, electron transport, and protein metabolism) along a gradient of N availability established by fertilization with NH_4_NO_3_ in different soils. Nelson et al. (34) showed that soil C and N contents explained the number of detected N pathways in the soil samples. Furthermore, they distinguished between N cycling specialists, encoding only a few N transformation processes, and N cycling generalists, encoding all N transformation processes (34). Graf et al. (29) reported that partial denitrifiers with *nirS* (cytochrome *cd*_1_ variant for nitrite reductase) often carried genes that encode nitric oxide (*norBC*) and nitrous oxide (*nosZ*) reductases and, thus, have the potential to do the complete denitrification pathway. This was in contrast to denitrifiers that carry *nirK* (copper-binding variant of nitrite reductase), which showed less cooccurrence with *norBC* and *nosZ* (29). Another metagenomic study revealed atypical *nosZ* sequences in soils (35). Furthermore, metagenomic investigations have improved our understanding of the microbial N network in many stratified ecosystems with an oxic-anoxic transition zone, such as those found in open oceans or estuaries (1–3, 36). This indicates that the framework of metagenomics-based research also provides a promising approach for investigating freshwater sediment N cycling.

**FIG 1:**
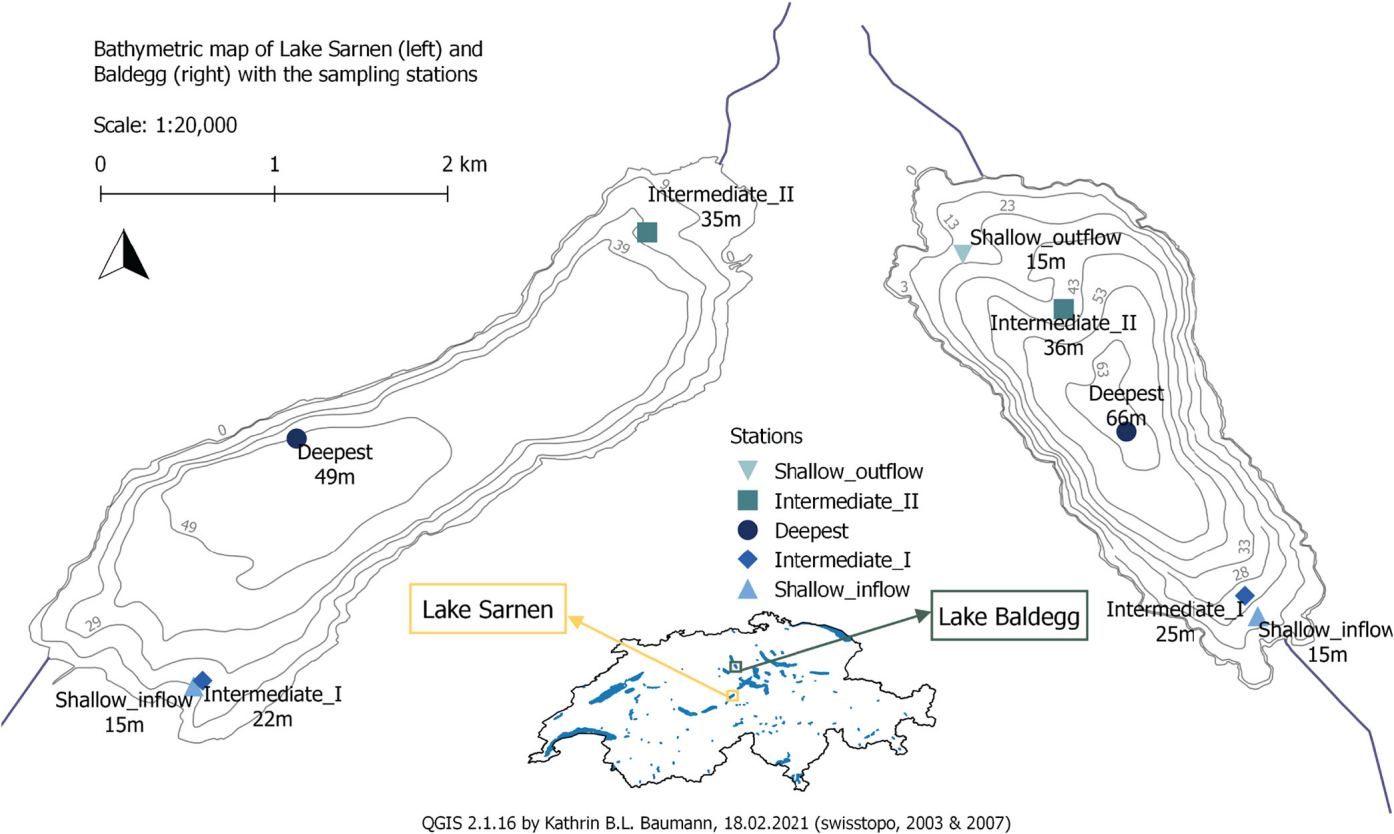
Sampling locations in Lake Sarnen (SAR) (left, yellow) and Lake Baldegg (BAL) (right, green), sampled in 2018 in March, May, August, and September. Pore water (0.25-cm resolution) and 16S rRNA gene sequencing (0.5-cm resolution) data were measured in all sampling points. DNA from the top 3 cm was pooled for metagenome analysis of the deepest station (SAR and BAL), Shallow_inflow (SAR), and Shallow_outflow (BAL) in all months. Sediment traps were deployed at the deepest point of each lake (dark blue); see Müller et al. (20).

In contrast to soils, ocean, and wastewater systems, few metagenomic studies of the N cycle have focused on freshwater lake sediments. However, lake sediments are central sites of N transformation and of crucial importance in regulating continental N exports. Here, we investigate the potential influence of lake trophic state on the N cycling genetic potential based on lake sediments of the eutrophic Lake Baldegg and the oligotrophic Lake Sarnen over seasonal and spatial scales (Fig. 1). We investigate lateral, vertical, and temporal variations in N-cycling microbial communities using high-throughput 16S rRNA gene amplicon sequencing and compare these to pore water fluxes of NO_3_^−^ and NH_4_^+^ recently reported for the same lakes by Müller et al. (20). We furthermore sequence shotgun metagenomes to provide a detailed view of N gene compositions and reveal the phylogenetic identities of microorganisms involved in N transformation reactions.

We hypothesize that the lakes’ contrasting N transformation processes are mediated by distinct microbial communities and that the genetic potential for microbial N cycling responds to environmental drivers such as the availability of different nutrients, including N species and OM inputs. Furthermore, we suggest that varying spatiotemporal pore water chemistry results in significant seasonal and spatial differences in the microbial N transformation potential and, thus, put into question the common practice of relying on single-core sampling to characterize whole lake ecosystems.

## RESULTS

### Microbial community composition and environmental drivers.

**FIG 2:**
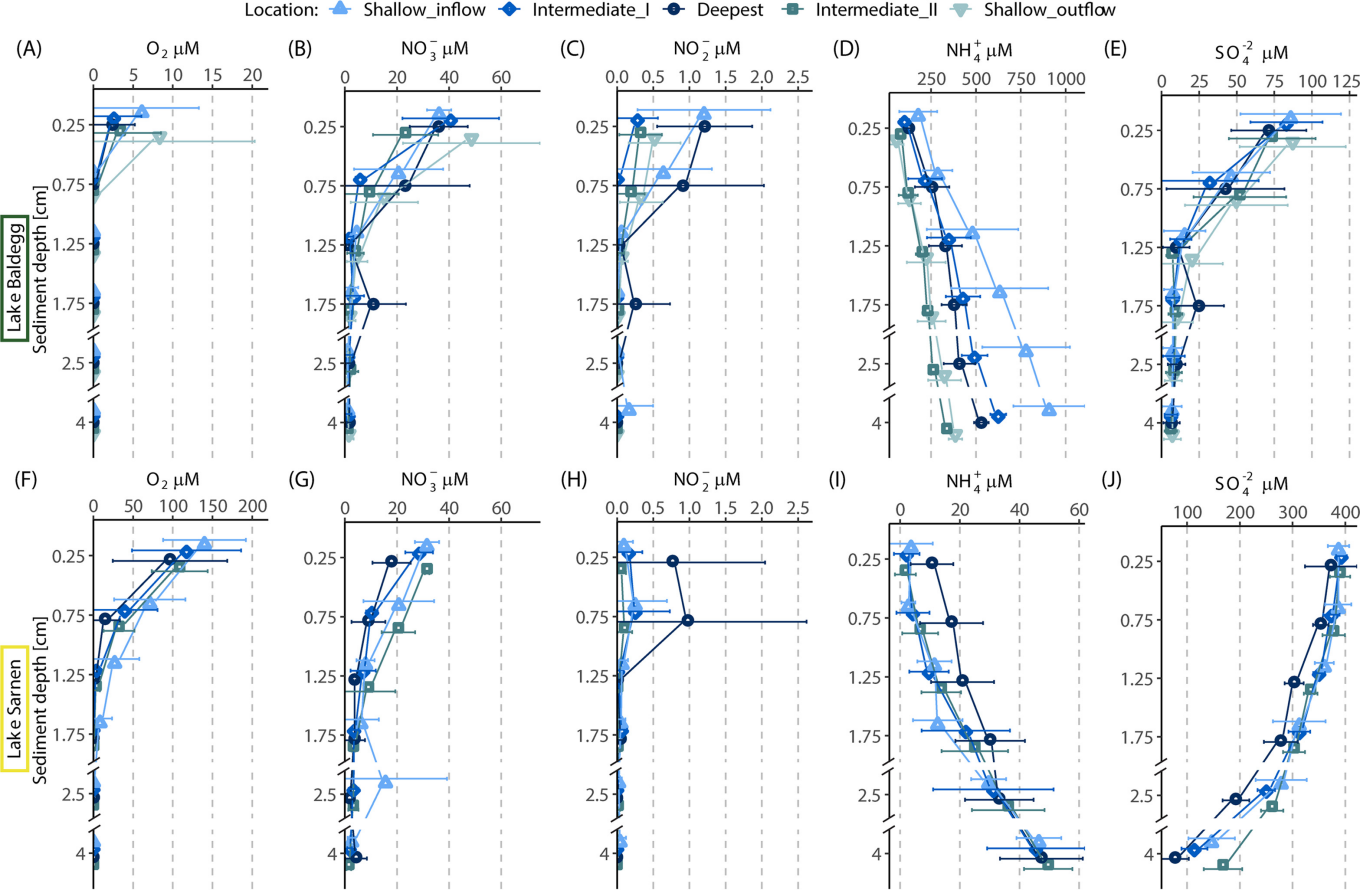
(A to E) Pore water and oxygen profiles of Lake Baldegg from the five different sampling locations (Shallow_inflow, 15 m; Intermediate_I, 25 m; deepest, 66 m; Intermediate_II, 36 m; and Shallow_outflow, 15m) and Lake Sarnen (F to J) from the four different sampling locations (Shallow_inflow, 15 m; Intermediate_I, 22 m; deepest, 49 m; and Intermediate_II, 35 m). Each graph shows the seasonal means (points; the points from each sediment depth were shifted slightly to prevent overlap and to distinguish them better) with standard deviations (vertical error bars) for each location within the sediment depth for the following chemical parameters: O_2_ (A and F), NO_3_^−^ (B and G), NO_2_^−^ (C and H), NH_4_^+^ (D and I), and SO_4_^2−^ (E and J).

Sediment pore water profiles from both lakes showed pronounced gradients of electron acceptors and reduced compounds, typical of freshwater lake sediment underlying an oxygenated water column. The sediment O_2_ concentrations at the sediment-water interface were ∼20 times higher in Lake Sarnen (maximum, 217 μM) than Lake Baldegg (maximum, 25 μM) (Fig. 2A and F). O_2_ also penetrated up to 4 times deeper in Lake Sarnen (<2 cm) than in Lake Baldegg (<0.75 mm) (Fig. 2A and F). In both lakes, the highest O_2_ concentrations were measured at the shallower station. O_2_ concentrations decreased in both lakes over the course of the year, with an average of 63 μM in March and 0 μM in September in Lake Baldegg and 181 μM in March and 67 μM in September in Lake Sarnen. The NO_3_^−^ levels in Lake Baldegg were slightly higher (maximum, 64 μM) than those of Lake Sarnen (maximum, 51 μM) (Fig. 2B and G). In Lake Sarnen, NO_3_^−^ penetrates deeper (∼4 cm), whereas NO_3_^−^ is depleted within the first 2 cm in Lake Baldegg. We did not observe any seasonal or spatial patterns for NO_3_^−^ (Fig. 2B and G). NO_2_^−^ concentrations generally remained below 2 μmol in both lakes (Fig. 2C and H). In Lake Baldegg, NO_2_^−^ was highest in the surface sediment (mean, 0.21 μM, in contrast to Lake Sarnen, where NO_2_^−^ peaked at 0.75 cm depth (mean, 0.13 μM) (Fig. 2C and H). NH_4_^+^ concentrations increased with sediment depth in both lakes, but in Lake Baldegg, concentrations (average, 337 μM) were over 10 times higher than those in Lake Sarnen (average, 21 μM) (Fig. 2D and I). The NH_4_^+^ concentration in Lake Baldegg decreased from inflow to outflow (Fig. 2D). In Lake Sarnen, the deepest station showed the highest NH_4_^+^ concentration in the upper sediment (Fig. 2I). Lake Sarnen had a higher concentration of SO_4_^2−^ than Lake Baldegg, with a mean SO_4_^2−^ concentration of 295 μM and 27.5 μM, respectively (Fig. 2E and J). The SO_4_^2−^ depletion curve in Lake Baldegg was steeper and stabilized at a low concentration below 2 cm sediment depth, in contrast to Lake Sarnen, where high SO_4_^2−^ concentrations were measured even at 4 cm sediment depth (Fig. 2E and J).

We observed distinct microbial community compositions in the two lakes. In Lake Sarnen, alpha diversity was significantly higher (Wilcoxon test, *P* < 0.001), with an observed total richness of 64,699 amplicon sequence variants (ASVs) (unfiltered) compared to Lake Baldegg, with 29,457 ASVs (see Fig. S1A and B in the supplemental material). The difference was robust to filtering out rare ASVs (taxa appearing less than five times in at least 10% of the samples), leaving 2,599 ASVs for Lake Baldegg and 6,124 ASVs for Lake Sarnen (Fig. S1A and B).

10.1128/msphere.01013-21.1 FIG S1 Overview of the alpha diversity measures (A and B) for the total microbial community composition based on the 16S rRNA of Lake Baldegg (green, 119 samples) and Lake Sarnen (yellow, 90 samples) and the significant differences between the alpha diversity of the two lakes. (C) Overview of the beta diversity measures for the whole microbial community composition based on the 16S rRNA of Lake Baldegg (green, 119 samples) and Lake Sarnen (yellow, 90 samples). All analyses were done in R with the vegan package by following the tutorial from the Küsel lab. Download FIG S1, EPS file, 1.4 MB. Copyright © 2022 Baumann et al. 2022 Baumann et al. https://creativecommons.org/licenses/by/4.0/ This content is distributed under the terms of the Creative Commons Attribution 4.0 International license.

**FIG 3:**
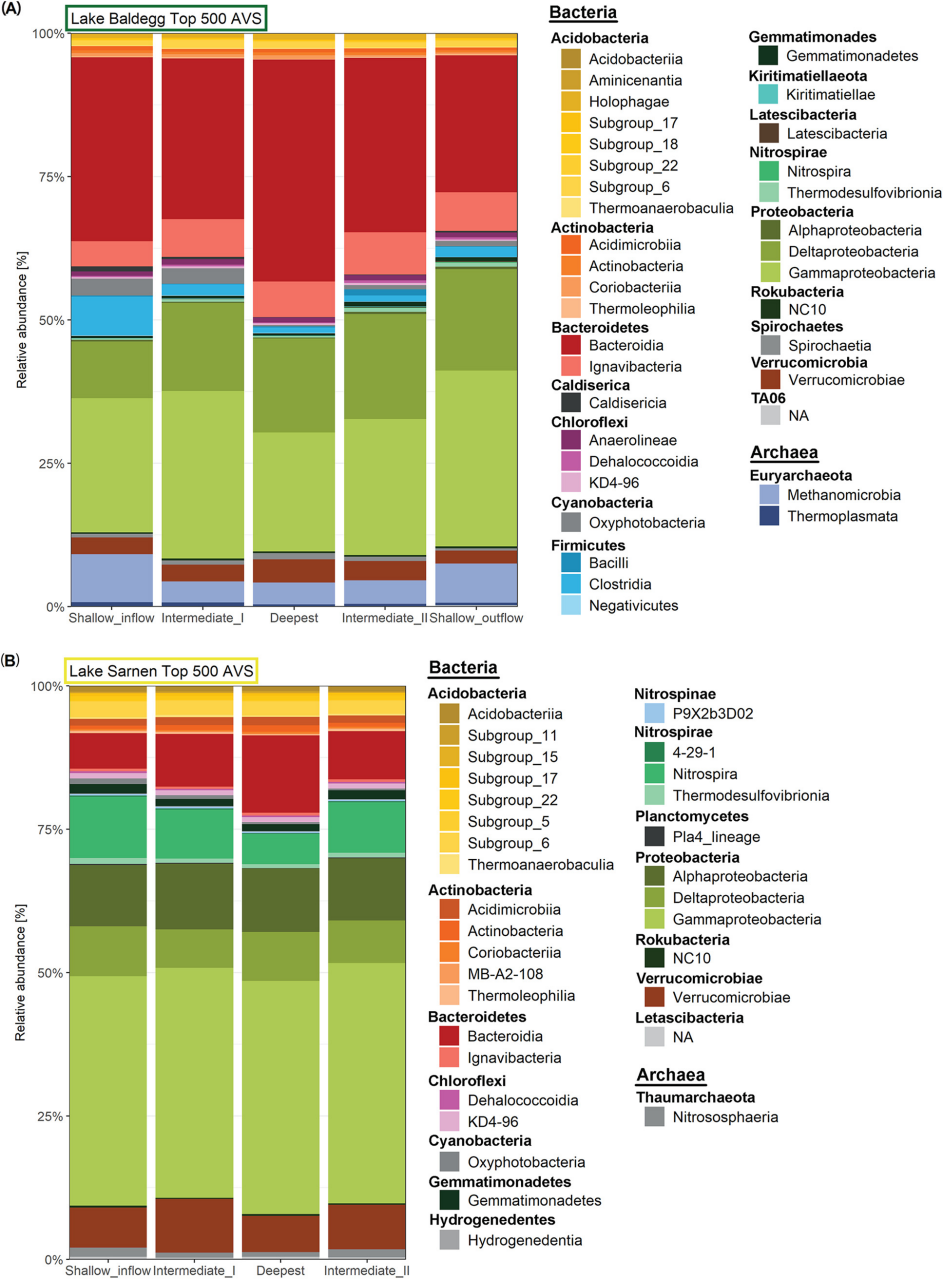
Microbial community composition of the top 500 ASVs summed at the class level in Lake Baldegg (top) and Lake Sarnen (bottom). Classes that belong to the same phylum are grouped together, and the phylum is written above each class group. The data shown are averaged over sediment depth and time for each station and separately for each lake.

The most abundant ASVs (top 500 ASVs) in Lake Baldegg were dominated by representatives of the phyla *Bacteroidetes* (represented by the main classes *Bacteroidia*, 31%, and *Ignavibacteria*, 6%), *Gamma* (26%)- and Deltaproteobacteria (16%), *Euryarchaeota* (mainly *Methanomicrobia*, 5%), and *Firmicutes* (mainly *Clostridia*, 2%) (Fig. 3A). In Lake Sarnen, the phylum *Proteobacteria* was also abundant, with a higher contribution of *Gamma*- (41%) and *Alphaproteobacteria* (11%) than Lake Baldegg. The *Bacteroidetes*, primarily of the class *Bacteroidia* (9%), were three times less abundant than in Lake Baldegg. The third most abundant phyla in Lake Sarnen were *Nitrospirae* (mostly *Nitrospira*, 8%), *Verrucomicrobiae* (8%), and *Acidobacteria* (5%) (Fig. 3B), which were only a minor component in Lake Baldegg. In Lake Sarnen, *Thaumarchaeota* (<1%) was the only archaeal phylum represented.

**FIG 4:**
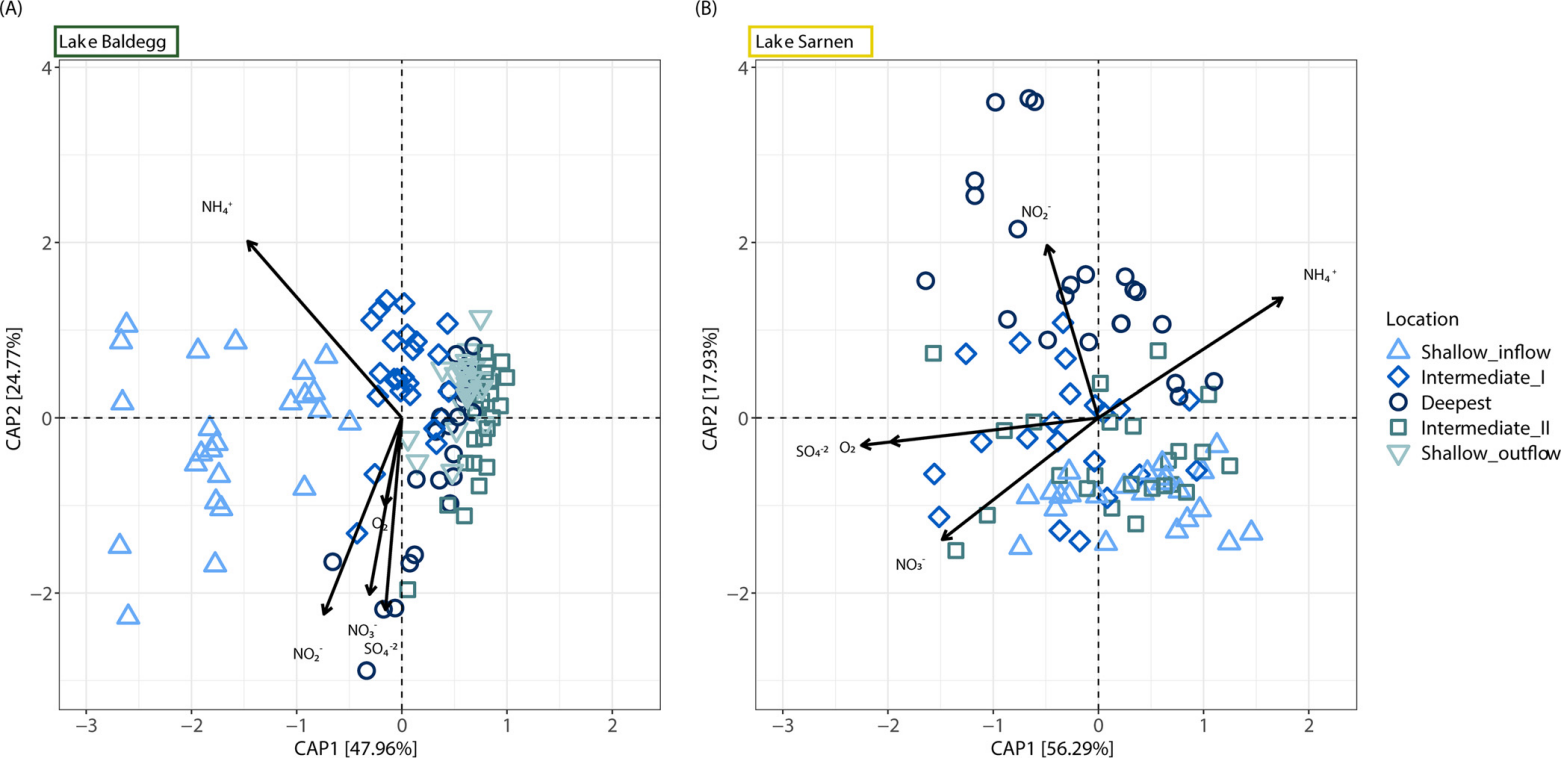
Constrained ordination biplots (redundancy analysis [RDA] on weighted UniFrac distance) of microbial community composition and the different environmental drivers (O_2_, NO_3_^−^, NO_2_^−^, NH_4_^+^ and SO_4_^2−^) for Lake Baldegg (A) and Lake Sarnen (B). The percentage of the variance explained for each component is labeled on the *x* and *y* axes. In Lake Baldegg, the two main components (CPA1 and CPA2) explained 72% of the variance in the microbial communities. In Lake Sarnen, the main components (CPA1 and CPA2) explain 74% of the variance in the microbial communities. The samples are colored and shaped by location.

Constrained ordination by RDA confirmed that community composition was significantly different in the two lakes (permutational analysis of variance [PERMANOVA], *P* < 0.001, *R*^2^ = 0.59) (Fig. S1C). The analysis also showed that beta-diversity patterns in the two lakes differed considerably (Fig. 4). In Lake Baldegg, the first ordination axis (CAP1, explained variance of 48%) reflects the location, and the second component (CAP2, explained variance of 25%) explains the microbial community composition differences over sediment depth (Fig. 4A and Fig. S2A and C). The microbial community composition significantly differed by sampling station (PERMANOVA, *P* < 0.001, *R*^2^ = 0.44) in Lake Baldegg, with stations generally sorting along axis 1 from inflow to outflow (Fig. 4A). The influence of sediment depth and sampling month on the community composition was also significant (PERMANOVA, *P* < 0.001), although with much lower explanatory power (*R*^2^ = 0.06 months and *R*^2^ = 0.05 sediment depth). In contrast, for Lake Sarnen, the first component reflected the microbial community composition shift with sediment depth (CAP1, explained variance of 56%), and the second component reflected community compositional differences by location (CAP2, explained variance of 18%), indicating fewer differences between locations than in Lake Baldegg (Fig. 4B). The community composition varied significantly between sampling stations in Lake Sarnen (PERMANOVA, *P* < 0.001, *R*^2^ = 0.21). The deepest station of Lake Sarnen revealed the most distinct community composition compared to the three other locations, where the two intermediate stations showed the most similar microbial community composition (Fig. 4B). As in Lake Baldegg, the community differences by sediment depth and sampling month were significant (PERMANOVA, *P* < 0.001) but with lower explanatory power (*R*^2^ = 0.09 sediment depth and *R*^2^ = 0.05 months) (Fig. S2B and D). RDA indicated that NH_4_^+^ concentration displayed the highest correlation with the observed changes in community composition in both lakes’ deeper sediments (Fig. S2A and B). In contrast, the other four environmental drivers (O_2_, NO_3_^−^, NO_2_^−^, and SO_4_^2−^) were drivers primarily for the microbial communities living in the surface sediment (Fig. S2A and B).

10.1128/msphere.01013-21.2 FIG S2 Constrained ordination biplots (redundancy analysis [RDA] on weighted UniFrac distance) of microbial community composition and the different environmental drivers (O2, NO_3_^−^, NO_2_^−^, NH4^+^, and SO_4_^2−^) for Lake Baldegg (A and C) and Lake Sarnen (B and D). The samples are colored by sediment depth (A and B) and month (C and D). All analyses were done in R with the vegan package by following the tutorial from the Küsel lab. Download FIG S2, EPS file, 0.1 MB. Copyright © 2022 Baumann et al. 2022 Baumann et al. https://creativecommons.org/licenses/by/4.0/ This content is distributed under the terms of the Creative Commons Attribution 4.0 International license.

### Microbial nitrogen transformation potential and possible key players.

**FIG 5:**
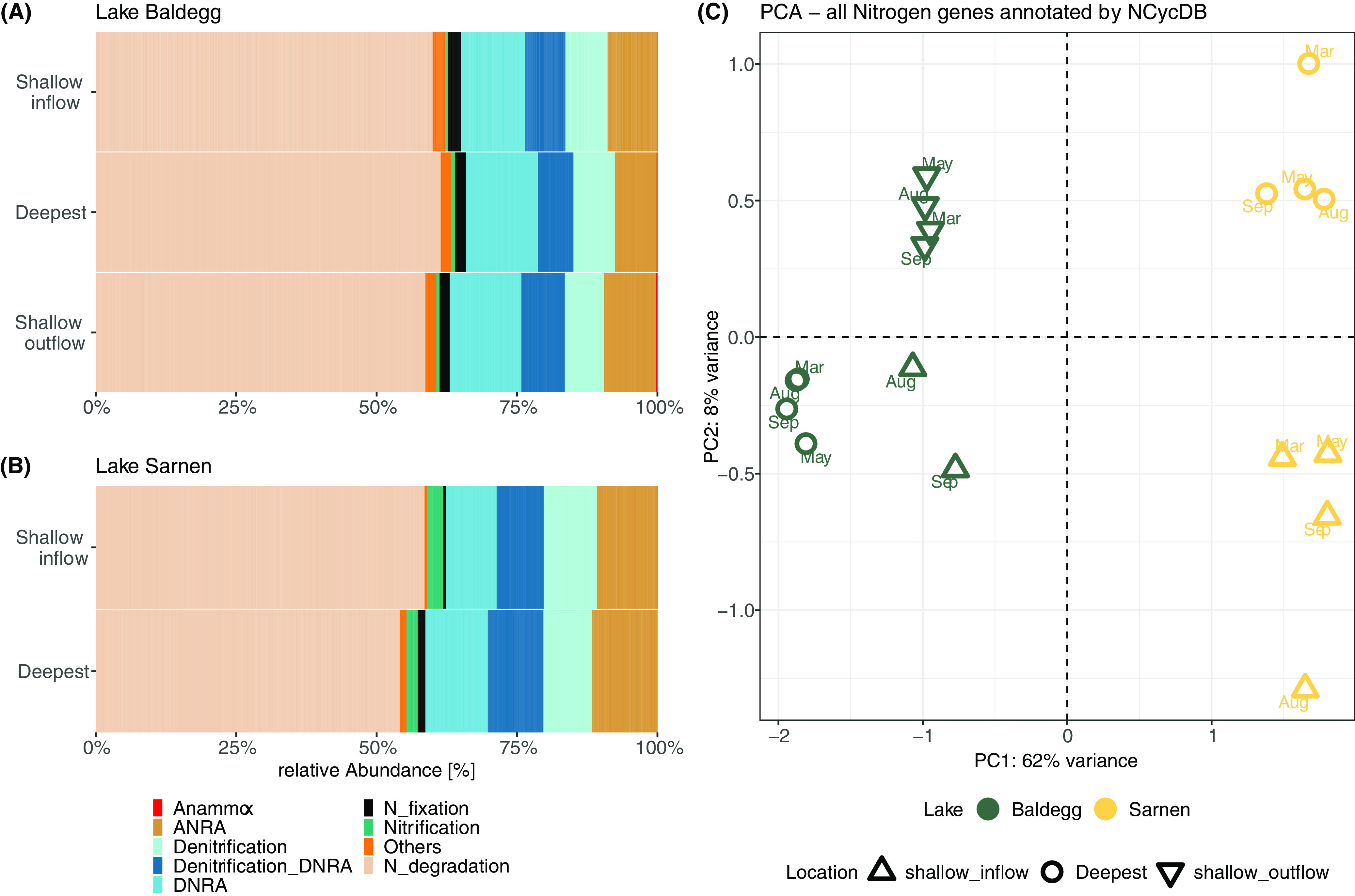
Nitrogen transformation gene distribution in Lake Baldegg (A) and Lake Sarnen (B) for each station summed over all seasons, grouped by the nitrogen transformation processes (Table 2). Nitrate reduction genes that cannot be assigned to either denitrification or DNRA are grouped together (Denitrification_DNRA). (C) The principal component analysis shows the nitrogen gene counts for each station, calculated with the DESeq2 package.

A total of 32,363 and 18,951 genes were indicative of N transformation processes in Lake Baldegg and Lake Sarnen, respectively (Table S2). Ordination of N transformation gene composition clearly distinguished lakes (PC1, 62%) and also locations (PC2, 8%) (Fig. 5C), whereas seasonal variations were comparatively minor (Fig. 5C). N gene abundance varied considerably, from 35,565 genes per million (gpm) for N degradation and biosynthesis in Lake Baldegg to 10 gpm for anammox in Lake Sarnen. The N gene abundance differed substantially between the lakes, especially for DNRA (Lake Baldegg, 7,638 gpm; Lake Sarnen, 4,716 gpm), N_2_ fixation (Lake Baldegg, 1,155 gpm; Lake Sarnen, 443 gpm), hydroxylamine reductase (*hcp*) (Lake Baldegg, 1,006 gpm; Lake Sarnen, 80 gpm), anammox (Lake Baldegg, 108 gpm; Lake Sarnen, 10 gpm), and nitrification (Lake Baldegg, 400 gpm; Lake Sarnen, 1,456 gpm) (Fig. 5A and Table S3). Denitrification (Lake Baldegg, 4,517 gpm; Lake Sarnen, 4,650 gpm) and nitrate reduction genes belonging to either DNRA or denitrification (Lake Baldegg, 4,347 gpm; Lake Sarnen, 4,336 gpm) occurred at similar abundances in both lakes (Fig. 5A and Table S3). We further analyzed methane-monooxygenase (*pmoABC*), a homologous gene family with high sequence similarity to *amoABC*. In both lakes, the *pmoABC* genes were less abundant than *amoABC* (Lake Baldegg, 105 gpm; Lake Sarnen, 379 gpm) (Table S3).

10.1128/msphere.01013-21.6 TABLE S2 Sequencing overview for the metagenomic data, with the number of raw reads, raw bases (G, giga), GC content, number of contigs (assembled sequences), number of contigs aligned to NCycDB, and number of total N genes (count N genes from NCycDB pipline) per sample (1). Download Table S2, XLSX file, 0.01 MB. Copyright © 2022 Baumann et al. 2022 Baumann et al. https://creativecommons.org/licenses/by/4.0/ This content is distributed under the terms of the Creative Commons Attribution 4.0 International license.

10.1128/msphere.01013-21.7 TABLE S3 Summary of all nitrogen cycle genes found in Lake Baldegg and Lake Sarnen from the different locations and months. The numbers are the sum of gpm (genes per million) per sample. The genes are clustered in the N transformation process they belong to. Download Table S3, XLSX file, 0.01 MB. Copyright © 2022 Baumann et al. 2022 Baumann et al. https://creativecommons.org/licenses/by/4.0/ This content is distributed under the terms of the Creative Commons Attribution 4.0 International license.

**FIG 6:**
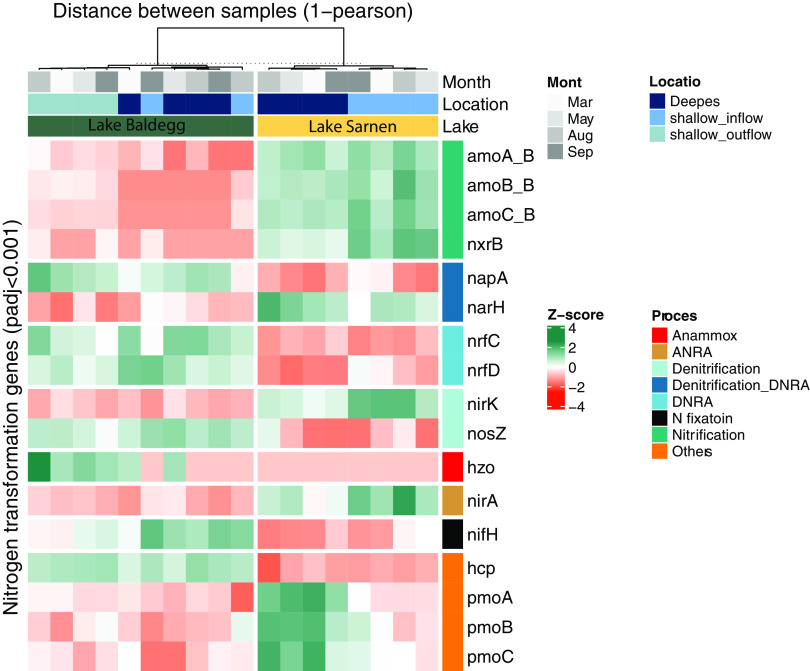
Heatmap of nitrogen gene abundances showing significant differences between Lake Baldegg (green) and Lake Sarnen (yellow) (alpha = 0.001; padj, adjusted *P* value with Benjamini-Hochberg method from DESeq2) clustered by lake and station (column clustering distance calculated using Pearson from the ComplexHeatmap package) in the columns and nitrogen process in the rows. The *z*-score of abundances was calculated for each gene individually to emphasize the differences.

**FIG 7:**
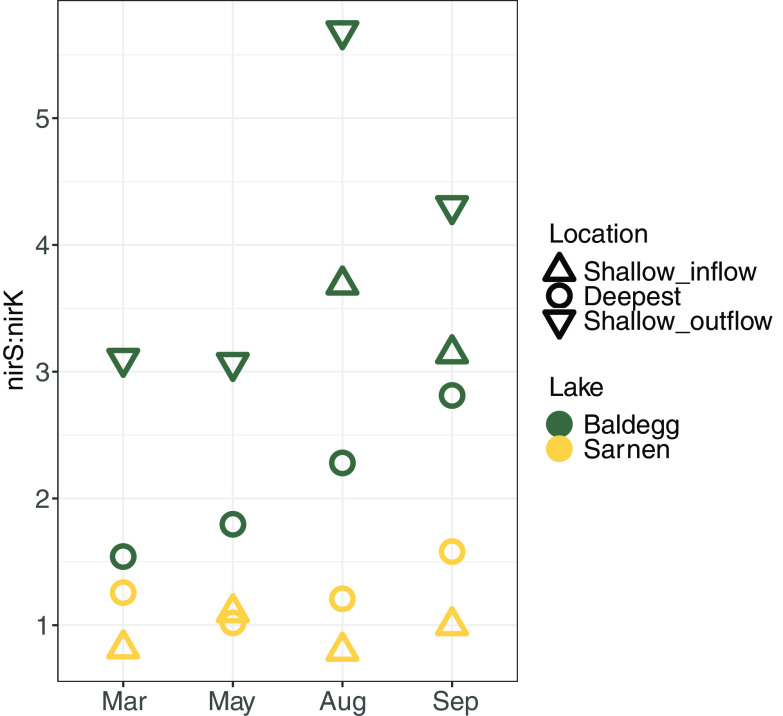
*nirS*:*nirK* ratio calculated from gpm values per lake (Baldegg, green; Sarnen, yellow), location (shallow inflow, triangle; deepest, circle; shallow outflow, rotated triangle), and month.

We found 17 genes involved in various N transformation pathways that had significantly different abundances between Lake Baldegg and Lake Sarnen (Deseq2, Wald test, adjusted *P* value [padj] of <0.001) (Fig. 6). In agreement with observations made for the microbial community composition (Fig. 4), we did not observe a distinct temporal clustering for N gene abundance data, but there was a clustering by sampling location evident in the heatmap (Fig. 6). We tested the spatial and temporal N transformation differences with DESeq2 using lakes plus station and lakes plus months as conditions. The results revealed 14 significantly different genes between the lakes and sampling location (*P* < 0.01) and 0 significantly different genes between the lakes and sampling time point (*P* < 0.01). Lake Sarnen had a significantly higher abundance of bacterial ammonia (*amoABC_B*) and nitrite-oxidizing (*nxrB*) genes than Lake Baldegg (Deseq2, Wald test, padj < 0.001), indicating a higher nitrification potential (Fig. 6). In Lake Baldegg, the shallow_outflow location showed a higher nitrification potential than the deep location (Fig. 6). Lake Baldegg had a significantly higher potential to reduce NO_3_^−^ via periplasmic nitrate reductase (*napA*) (Deseq2, Wald test, padj < 0.001), whereas Lake Sarnen showed a significantly higher abundance for a different nitrate reductase gene, *narH* (Deseq2, Wald test, padj < 0.001) (Fig. 6). Two representative genes that encode NO_2_^−^ reduction to NH_4_^+^ in the DNRA pathway (*nrfC* and *nrfD*) were significantly higher in Lake Baldegg than in Lake Sarnen (Deseq2, Wald test, padj < 0.001) (Fig. 6). Among genes that encode steps of denitrification, *nirK* was significantly more abundant in Lake Sarnen, especially in the shallow stations (Deseq2, Wald test, padj < 0.001), while *nosZ* was significantly more abundant in Lake Baldegg (Deseq2, Wald test, padj < 0.001). *nirS* had similar abundances in both lakes, resulting in a higher *nirS*:*nirK* ratio for Lake Baldegg than Lake Sarnen, especially in the shallower stations (Fig. 7). Anammox genes were significantly more abundant in Lake Baldegg (Deseq2, Wald test, padj < 0.001) and were highest in the shallow outflow location (Fig. 6). The gene encoding the assimilatory NO_3_^−^ reduction to NO_2_^−^ (*nirA*) was significantly more abundant in Lake Sarnen than Lake Baldegg (Deseq2, Wald test, padj < 0.001) (Fig. 6). In contrast, Lake Baldegg showed a significantly higher abundance of *nifHK*, which encodes nitrogenase, the central enzyme for N_2_ fixation. The N_2_ fixation potential was especially high in the deep and shallow inflow stations (Deseq2, Wald test, padj < 0.001) (Fig. 6). The hydroxylamine reductase gene *hcp* was significantly more abundant in Lake Baldegg, whereas the genes encoding methane oxidation (*pmoABC*) were significantly more abundant in Lake Sarnen (Deseq2, Wald test, padj < 0.001).

About 47% of the significant N transformation genes could be taxonomically assigned to at least the class level. The results suggest that different key players dominate N cycling in each lake (Table 1 and Table S5). Overall, most represented among nitrogen-cycling taxa were members of the *Gammaproteobacteria* (2,950 gpm), followed by Deltaproteobacteria (1,462 gpm), *Bacteroidetes* (720 gpm), and *Nitrospirae* (638 gpm). The most abundant nitrification genes (*nxrB* and *amoABC*) were assigned to the nitrite-oxidizing bacteria (NOB) of the phylum *Nitrospirae*, with Lake Sarnen having a higher abundance (300 gpm) than Lake Baldegg (9 gpm) (Table 1). In contrast, the ammonia-oxidizing bacteria (AOB) of the order *Nitrosomonadales* (*Gammaproteobacteria*) were represented in both lakes. The nitrate and nitrite reductase genes from ANRA, DNRA, and Dentrification_DNRA were assigned to a phylogenetically diverse group of microbes (Table 1). In Lake Sarnen, the most abundant phyla carrying *nirK* were *Nitrospirae* (157 gpm) and *Chloroflexi* (113 gpm). In contrast, in Lake Baldegg, *nirK* sequences were mainly assigned to *Gammaproteobacteria* (86 gpm) and *Bacteroidetes* (52 gpm) (Table 1). The phylum most frequently assigned to *nirS* was *Gammaproteobacteria*, representing 448 gpm in Lake Baldegg and 211 gpm in Lake Sarnen, respectively. The most abundant phyla containing the nitrous oxide-reducing denitrification gene (*nosZ*) in Lake Baldegg and Lake Sarnen were *Bacteroidetes* (140 and 54 gpm) and *Gammaproteobacteria* (158 and 49 gpm). Nitrogenase genes (*nifK*) in Lake Baldegg were often assigned to *Euryarchaeota* (173 gpm).

**TABLE 1:** Overview of the mapping rates of the N removal genes to the Kaiju database to reveal possible key players (top 12 phyla) for the significantly different gene families plus *nirS*^*a*^

Organism(s)	Nitrification for *amoABC*, *nxrB*		Denitrification (DNRA)				DNRA for *nrfCD*		Denitrification						Anammox for *hzo*, *hszA*		ANRA for *nirA*		N_fixation for *nifHK*		Others for *hcp*	
			*napA*		*narH*				*nirK*		*nirS*		*nosZ*									
	BAL	SAR	BAL	SAR	BAL	SAR	BAL	SAR	BAL	SAR	BAL	SAR	BAL	SAR	BAL	SAR	BAL	SAR	BAL	SAR	BAL	SAR
*Acidobacteria*			2	12	6	6	**119**	18	2	12	2	14	13	11	2		2	1	6		4	
*Actinobacteria*			5		6	7	13	4	6	8	2	1	4	3			3	6			1	
*Alphaproteobacteria* ^*b*^			2	4	2	34	25	9	7	22	3	8	5	19			8	32	11	2	2	
*Bacteroidetes*			**19**	1	5		**364**	20	**52**	8	15		**140**	54	4		7	4	16	3	8	
*Chlorobi*			1				**106**	11	10		6		22									
*Chloroflexi*			17	14	3		26	38	31	113	15	1	51	37			6	11	2	4	11	
*Deltaproteobacteria* ^*c*^	**71**	12	**217**	19	**84**	11	**526**	102	14	5	**116**	25	**115**	37			**20**		**74**	9	**285**	20
*Euryarchaeota*					1		20		3		10	4	1				8		**149**		31	
*Gammaproteobacteria* ^*d*^	**28**	45	**105**	63	**86**	142	**669**	432	**86**	87	**448**	211	**158**	49			119	106	**71**	37	8	
*Nitrospirae*	9	300	3	9			38	10	1	157		65					1	30	1	13	1	
*Planctomycetes*			1	6			2		9	22	16		13	5	**11**				6		8	3
*Verrucomicrobia*			27	6	10		26	39	10	45	11	5	14	32	1		7	17	5		6	

a The numbers are the sum of gpm per gene per lake and phyla. The most abundant phyla are highlighted in boldface for Lake Baldegg and underlining for Lake Sarnen. The most abundant classes of the *Proteobacteria* are listed in the footnotes below. The full list of *Proteobacteria* orders per gene can be found in Table S5. Note that *Gammaproteobacteria* include *Betaproteobacteria.*

b The most assigned orders of *Alphaproteobacteria* were *Caulobacterales* (most often assigned in Lake Sarnen), *Rhizobiales*, *Rhodobacterales* (most often assigned in Lake Sarnen), and *Rhodosprillales.*

c The most abundant orders of Deltaproteobacteria were *Desulfobacterales*, *Desulfuromonoadales*, and *Syntrophobacterales.*

d The most often assigned *Gammaproteobacteria* (including *Betaproteobacteria*) orders were *Acidiferrobacterales* (most often assigned in Lake Sarnen), *Aeromonadales* (most often assigned in Lake Sarnen), *Chromatiales*, *Enterobacterales* (most abundant in Lake Baldegg), *Methylococcales*, *Tiotrichales* (most abundant in Lake Baldegg), *Burkolderiales*, *Nitrosmonadales*, and *Rhodocyclales.*

10.1128/msphere.01013-21.9 TABLE S5 Overview of the mapping rates of the N removal genes to Kaiju database of *Proteobacteria* (top 12 phyla) for the significantly different gene families, plus *nirS*. The numbers are the total sum of gpm per genes per lake and phylum. The most abundant are highlighted in green for Lake Baldegg and yellow for Lake Sarnen. We listed *Betaproteobacteria* here separately, as Kaiju assign them separately and does not have the SILVA132 database assignment. In the paper, *Beta*- and *Gammaproteobacteria* are summed (92, 93). Download Table S5, XLSX file, 0.02 MB. Copyright © 2022 Baumann et al. 2022 Baumann et al. https://creativecommons.org/licenses/by/4.0/ This content is distributed under the terms of the Creative Commons Attribution 4.0 International license.

## DISCUSSION

### Distinct microbial communities and pore water chemistry.

The pore water chemistry in the eutrophic and oligotrophic lake sediments studied was clearly a driver of microbial community composition. However, we observed that while the chemical sediment pore water signature was seasonally driven to a considerable extent (20), the microbial community composition was mainly affected by location (Fig. 4; see also Fig. S2 in the supplemental material). The sediment pore water profiles of eutrophic Lake Baldegg indicated a high availability of inorganic N (Fig. 2). The rapid depletion of O_2_, NO_3_^−^, NO_2_^−^, and SO_4_^2−^ and the increasing concentration of NH_4_^+^ with sediment depth indicated a high aerobic and anaerobic respiration activity, in agreement with previous reports from Lake Baldegg (20, 37, 38). Lake Sarnen, in contrast, had sediment pore water profiles that were similar to those of other oligotrophic lakes, e.g., Lakes Lucerne (37), Brienz, and Thun (38), and lower concentrations of inorganic N than in Lake Baldegg. The O_2_ concentrations and penetration depth, as well as the SO_4_^2−^ concentrations, were higher in Lake Sarnen, indicating lower respiratory activity (Fig. 2). Overall, the pore water data confirmed that the lake sediments chosen for this study were suitable endmembers of the oligotrophic to eutrophic spectrum of lakes with aerobic water columns in Switzerland.

The two lakes share many microbial taxa. The most abundant phyla occurring in both lakes (Fig. 3) have previously been reported to also dominate in other lake sediments (25, 39–44). Nonetheless, the alpha and beta diversity analysis indicated a very distinct community composition (Fig. 4 and Fig. S1 and S2). We hypothesize that the significantly higher species richness in Lake Sarnen is explained by a greater number of fastidious oligotrophic microbes due to the low nutrient content. In contrast, the high nutrient abundance in Lake Baldegg appears to favor a smaller number of competitive, copiotrophic microbes. Similar observations have been made for the planktonic diversity in oligotrophic mountain lakes (45–48). Further studies are required to test this hypothesis.

The microbial community composition changes over sediment depth are directly linked to the sediment's redox cascade (Fig. S2A and B). For example, the high abundance of *Nitrospirae* in the surface sediment, especially in Lake Sarnen (Fig. S4), can be linked to low NH_4_^+^ and high O_2_ concentrations. *Nitrospirae* is a known aerobic NOB occurring at low NH_4_^+^ concentrations, as in Lake Sarnen (49). The same habitat preferences were reported for *Nitrososphaeria*, ammonia-oxidizing archaea (AOA) (50), which were only found in Lake Sarnen sediments. The absence of NOB and AOA in Lake Baldegg sediments can be explained by the rapid O_2_ depletion and high NH_4_^+^ concentrations in the first few millimeters. In contrast, the Deltaproteobacteria, among which many anaerobic sulfate-reducing bacteria are found (51), and *Methanomicrobia* (anaerobic *Euryarchaeota*) became more dominant with increasing sediment depth in Lake Baldegg (Fig. S3). Similar trends in Lake Baldegg were reported in 2016 by Han et al. (39).

10.1128/msphere.01013-21.3 FIG S3 Spatial (columns), sediment depth (rows), and seasonal (*x* axis) distribution of the top 500 ASVs (relative abundance) of Lake Baldegg. Classes that belong to the same phylum are grouped together, and the phylum is written above each class group. The data show the sediment depth and temporal average for each station per lake separately. The ASVs were retrieved with DADA2 and assigned using the SILVA132 database. Download FIG S3, TIF file, 2.3 MB. Copyright © 2022 Baumann et al. 2022 Baumann et al. https://creativecommons.org/licenses/by/4.0/ This content is distributed under the terms of the Creative Commons Attribution 4.0 International license.

10.1128/msphere.01013-21.4 FIG S4 Spatial (columns), sediment depth (rows), and seasonal (*x* axis) distribution of the top 500 ASVs (relative abundance) of Lake Sarnen. Classes that belong to the same phylum are grouped together, and the phylum is written above each class group. The data show the sediment depth and temporal average for each station per lake separately. The ASVs were retrieved with DADA2 and assigned using the SILVA132 database. Download FIG S4, TIF file, 2.7 MB. Copyright © 2022 Baumann et al. 2022 Baumann et al. https://creativecommons.org/licenses/by/4.0/ This content is distributed under the terms of the Creative Commons Attribution 4.0 International license.

Within lakes, locations differed considerably. We found the most distinctive microbial community composition at the shallow inflow location of Lake Baldegg, which could be explained by high nutrient and organic matter input from the tributary. The deeper and intermediate stations also contained distinct communities. One explanation for spatial differences can be sediment focusing. Both lakes have rather steep shorelines (Fig. 1), which can lead to sediment movements toward the deeper parts of the lake in a process called sediment focusing (52). The overall greater community similarity among samples from different locations in Lake Sarnen could be explained by the influence of its alpine river tributaries, which can have very high flow rates and transport large amounts of terrestrial material (53). During storm events, we measured an increase in sedimentation rates from ∼8 g m^−2^ day^−1^ to 556 g m^−2^ day^−1^ in June 2017 and 112 g m^−2^ day^−1^ in January 2018 at the deepest station ∼2 km from the inflow (20). Therefore, we assume that the larger input of terrestrial OM and river sediments and its wide distribution in the lake contribute to lower local niche diversity in Lake Sarnen compared to Lake Baldegg.

Seasonal changes in microbial community composition were only apparent among surface sediment samples, in which *Cyanobacteria* became more abundant during August and September, mainly in Lake Baldegg (Fig. S3) and less strongly in Lake Sarnen (Fig. S4). This finding likely reflects the seasonal sedimentation of biomass from cyanobacterial blooms in the surface water in late spring and summer, which has also been observed in sediment trap samples (20).

### Microbial nitrogen transformation potential.

**FIG 8:**
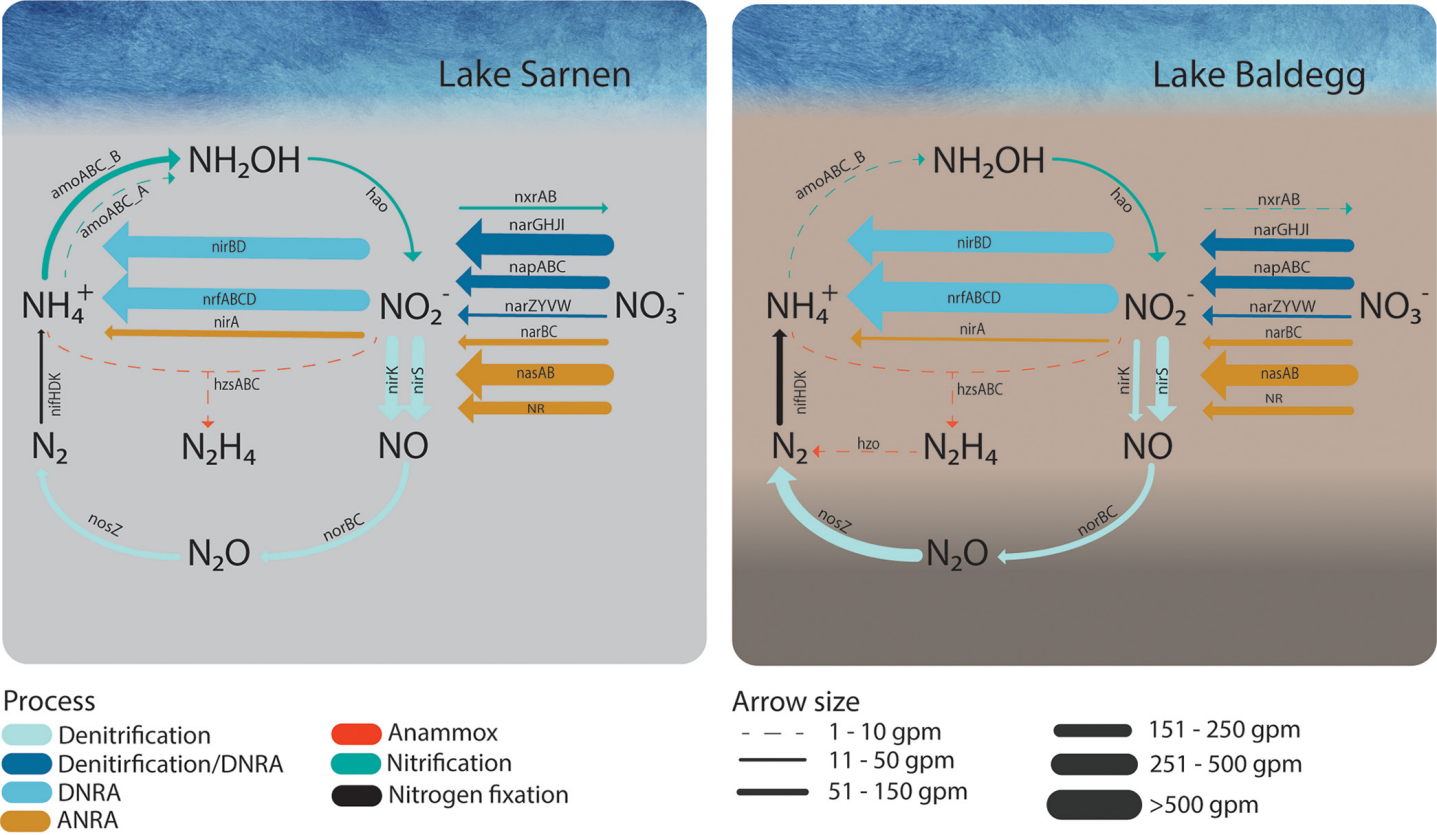
Conceptual overview of the N transformation processes by gene families in Lake Sarnen (left) and Lake Baldegg (right). The arrow thickness indicates the average gpm per lake for the different transformation processes. The gene families are colored in the different transformation processes they belong to nitrification (turquoise), ANRA (brown), denitrification_DNRA nitrate reductase genes (dark blue), DNRA (bright blue), denitrification (bright turquoise), anammox (red), and nitrogen (N_2_) fixation (N_fixation in black).

Our metagenomic analysis revealed that both lakes harbor microbial communities with the potential to perform almost all known N transformation processes (Fig. 8). This is in agreement with metagenomic studies of various ecosystems recovering the genetic potential for most, if not all, N transformation pathways (26, 54–57). Despite the overall similarities in the N cycling potential, ecosystems harbor unique N transforming microbial communities forming complex networks (1). Our work showed that this is also true for different freshwater lake systems and even for different sediment patches. The N gene composition and taxonomic data indicated spatial differences in the microbial community composition and N transformation potential between and within both lakes. This is in agreement with previous observations of a local difference in denitrification rates in lake sediments (25). This finding emphasizes the importance of including local differences of microbial community composition and function in lake sediment studies.

We saw little evidence for seasonal variation in the sediment microbial communities or their N cycling potential. This indicates that the assembly of the largely sedentary microbial community in the sediment, together with its N transformation potential, plays out over longer time scales, and these communities do not adapt rapidly to minor seasonal fluctuations in environmental conditions. This does not exclude short-term responses of activity, which can only be clarified by N transformation rate measurements and by applying methods that characterize the active microbial community, e.g., metatranscriptomics or proteomics.

### Nitrogen inputs: organic N transformation and N_2_ fixation.

Organic N transformation (N_degradation) was highly abundant in both lakes, indicating that microbes can degrade organic N compounds in the OM to produce reactive N (Fig. 5). The ecology of organic N mineralization is not well characterized. Our results suggest that this function is more widespread among sediment microbial communities than the more specialized respiratory N transformations. Another way for the ecosystem to get reactive N is N_2_ fixation. In the water column of lakes, N_2_ fixation is a well-known process (3, 22, 36, 58). Nitrogenase genes were found in all samples in this study (Fig. 5). However, despite our observation of seasonal deposition of cyanobacteria, cyanobacterial nitrogenase genes were not identified in the sediment of Lake Baldegg. Instead, the abundant N_2_ fixation genes in Lake Baldegg were mainly assigned to methanogenic *Euryarchaeota* (*Methanomicrobiales*) and *Proteobacteria* (Table 1). Methanogenic *Euryarchaeota* has been reported from wetland soils, where they actively transcribed *nifH* genes (59). This leads to the assumption that Lake Baldegg's sediment microbial community could respond to N scarcity. However, given the high NH_4_^+^ concentration in the sediments, this is unlikely to represent an important way to obtain reactive N under the current conditions. Further research would be required to verify this assumption.

### Nitrification.

Lake Sarnen's higher nitrification potential compared to Lake Baldegg is expected due to its higher O_2_ concentration and penetration depth, since nitrification is an aerobic process. Similar results have been reported for river freshwater sediments (60). The shallower O_2_ penetration depth in Lake Baldegg sediments likely constrains the development of an abundant nitrification community, which is supported by our rate measurements (Callbeck et al., unpublished). Here, instead, nitrification dominates in the overlaying water where O_2_ is more readily available (Callbeck et al., unpublished). Lake Baldegg showed the highest abundance for *Nitrosomonadales* among identified AOB. This taxon mediates partial nitrification by oxidizing NH_4_^+^ to NO_2_^−^ (encoded by *amoABC*) and can thrive under low O_2_ concentrations (1, 23). *Nitrospirales*, well-known NOBs (1, 36, 50, 60), were the main taxa assigned to *nxrB* in Lake Sarnen (Table 1). Interestingly, *amoABC_B* genes were also assigned to *Nitrospirales* in Lake Sarnen, indicating the potential for comammox (30, 31, 61). Comammox *Nitrospira* organisms have been reported from wetlands, agricultural and forest soil, wastewater treatment plants, drinking water systems (62), and sediments from Lake Superior (25). To test the hypothesis that comammox *Nitrospira* are abundant in Lake Sarnen, we mapped the bacterial *amoABC* genes against four comammox *Nitrospira* and two noncomammox *Nitrospira* genomes (30, 61, 63, 64). We found *amoABC* and *nxrB* genes from Lake Sarnen mapping to the comammox *Nitrospira* genomes but not those from Lake Baldegg, which supports our hypothesis (Table S4). Unfortunately, due to insufficient sequencing depth, we were unable to retrieve enough high-quality metagenome-assembled genomes (MAGs) to confirm the presence of comammox *Nitrospira*. Hence, at this time, we cannot provide further evidence that comammox contributes to NH_4_^+^ oxidation in Lake Sarnen sediments.

10.1128/msphere.01013-21.8 TABLE S4 Mapping results (number and percentage of reads) of the *amoABC* and *nxrB* genes against 4 comammox *Nitrospira* genomes, Ca.N.nitrosa (SAMEA3638995), and Ca.N.nitrificans (SAMEA3638996) (2), Ca.N.inopinata (SAMEA3560316) (3), and *Nitrospira* sp. strain GA0074138 (SAMN04254488) (4), and 2 non-comammox *Nitrospira* genomes, *N. defluvii* (SAMEA2272290), and *N. moscoviensis* (SAMN03702441) (5). The genes were mapped against the genomes using bbmap (minid=default (0.76), 0.85, 0.9) (version 38.49) (6). Download Table S4, XLSX file, 0.01 MB. Copyright © 2022 Baumann et al. 2022 Baumann et al. https://creativecommons.org/licenses/by/4.0/ This content is distributed under the terms of the Creative Commons Attribution 4.0 International license.

### NO_3_^−^ and NO_2_^−^ reduction processes.

Interestingly, we measured lower rates for DNRA than for denitrification in both lakes (Callbeck et al., unpublished), even though DNRA genes were more abundant than denitrification genes (Table S4). It seems that many microbes in these lakes have the capacity to perform DNRA if conditions are favorable for this process but generally rely more on denitrification. This is probably because denitrification has a higher energy yield than DNRA (36). Many environmental variables play a role in the competition between denitrification and DNRA. For example, higher input of labile organic carbon (C), an increasing C-N ratio, the ratio of electron donors to electron acceptors, and sulfide concentrations are all known to affect the balance between these processes (18, 27, 36, 65–67). The more balanced ratio of denitrification to DNRA activity in Lake Sarnen measured by Callbeck et al. (unpublished) may be a consequence of the higher C:N ratios or the presumably higher sulfide levels from sulfate reduction, both of which can promote DNRA (18, 36). On the other hand, inhibition of DNRA due to high NO_3_^−^ concentrations has been observed (65), which could explain the low DNRA rates in Lake Baldegg. However, it is not yet fully understood what conditions favor a switch from denitrification to DNRA in lake sediments (36).

There are other processes potentially competing for NO_2_^−^, such as anammox. Callbeck et al. (unpublished) measured low anammox rates relative to denitrification rates in the deepest station of Lake Baldegg. In contrast, in Lake Sarnen, the anammox rates in the surface sediment were as high as the denitrification rates. Interestingly, we could find some but not the complete set of anammox genes in Lake Sarnen. This suggests a low abundance of anammox bacteria, confirmed by the low abundance of *Planctomycetes* (the phylum containing all known anammox bacteria) in both lakes (Lake Baldegg, 0.01%; Lake Sarnen, 0.1% from all 16S rRNA sequences). However, a low abundance of anammox bacteria does not mean that anammox is not an active process. There have been reports of other aquatic ecosystems with high anammox rates and low abundance of *Planctomycetes* (11, 15, 68, 69). Furthermore, Crowe et al. (28) stated that anammox might be an important N loss process (up to 50% of the N loss in Lake Superior) for freshwater environments with low productivity, such as high-latitude lakes. However, more research is needed to resolve the role and activity of anammox in the oligotrophic Lake Sarnen.

### Denitrification.

The higher abundance of *nosZ* in Lake Baldegg compared to Lake Sarnen, especially at the deep station (Fig. 6), agrees with the reported higher denitrification rates and N removal rates in Lake Baldegg (20 and Callbeck et al., unpublished). Graf et al. (29) found that *nosZ* genes have a higher cooccurrence with *nirS* than *nirK*, suggesting that *nirS* denitrifiers are more likely to be capable of complete denitrification. Our observation of a higher *nirS*:*nirK* ratio in Lake Baldegg compared to Lake Sarnen suggests that the capability for complete denitrification is more important in this lake. *nirK* denitrifiers are reported to respond differently to environmental gradients than *nirS* denitrifiers and occupy a different ecological niche (29). Our results allow the formation of a hypothesis according to which lakes with higher trophic status harbor more denitrifiers with the *nirS* gene and the capacity for complete denitrification, while oligotrophic lakes provide an ecological niche for *nirK* denitrifiers. Thus, further studies should determine whether the *nirS*:*nirK* ratio differs in lakes with different trophic states.

### Nitrogen removal in a eutrophic and oligotrophic lake from the microbe's perspective.

Our data show that the trophic state did not impact the two lakes' overall microbial N transformation potential. However, internal N recycling through nitrification (including comammox), DNRA, and anammox appeared to be more important in the oligotrophic Lake Sarnen than in the eutrophic Lake Baldegg. In addition, the key players involved in N cycling clearly differ. Our findings are reflected in different N transformation rates and N removal efficiencies in the lakes studied. Müller et al. (20) calculated a much higher N removal rate (NRR) (20 ± 6.6 g N m^−2 ^year^−1^) and N removal efficiency (NRE; 66%) for Lake Baldegg compared to Lake Sarnen, with an NRR of 3.2 ± 4.2 g N m^−2 ^year^−1^ and an NRE of 33%. In agreement with that study, Callbeck et al. (unpublished data) measured more than 15 times higher denitrification rates in Lake Baldegg sediments (7.7 g N m^−2 ^year^−1^) than in Lake Sarnen (0.5 g N m^−2 ^year^−1^). Low denitrification activity in oligotrophic lakes is a known phenomenon reported from the Laurentian Great Lakes (25). This is because oligotrophic lakes might have a microbial N activity threshold and cannot achieve higher denitrification rates even under replete NO_3_^−^ concentrations (25). The higher denitrification rates in Lake Baldegg were linked to higher *nirS*:*nirK* ratios. Furthermore, the higher total organic carbon (TOC) input in Lake Baldegg stimulates the microbial N cycling as TOC acts as an electron donor for various N reduction pathways in organotrophic N reducing reactions, such as denitrification (1, 18, 25, 36). Additionally, we found that higher NO_3_^−^ concentrations in Lake Baldegg enhanced denitrification while inhibiting DNRA (70). Both mechanisms could explain the lower denitrification activity in Lake Sarnen. The considerable diversity and abundance of N transformation genes in the sediment surface layer show the tight connection of the various processes as reported in other studies focusing on the coupling of nitrification and denitrification in sediments (19). The combination of high TOC deposition, high NO_3_^−^ concentrations, and the artificial aeration of Lake Baldegg appears to create an optimal ecological niche for denitrifying bacteria. Lake Sarnen, in contrast, is less efficient in terms of N removal, but as an oligotrophic lake, it nevertheless exports little N with the outflow. Although less efficient at removing N, the oligotrophic Lake Sarnen harbors a high microbial diversity with important ecosystem functions.

### Conclusions.

Lake sediments were shown to harbor the genetic potential for all considered nitrogen cycle processes but in variable abundance, diversity, and performed by distinct microbial networks. Judging from the contrasting results obtained from the lakes studied here, the microbial potential for N transformation in lake sediments can differ considerably in individual freshwater lakes and varies with location within each lake. We have demonstrated that a detailed quantitative description of the N functional genes and potential microbial key players in lake sediments can be obtained through assembly-based metagenomic analysis. This microbial N gene potential forms the foundation of N removal on the ecosystem scale (20) and the specific N transformation rates of sediments (Callbeck et al., unpublished). N transformation potential in lake sediments clearly reflects the environmental conditions (e.g., availability of electron acceptors, OM input, etc., most of which are at least partly controlled by the lakes’ trophic state). This results in distinct microbial communities with a different representation of the processes linked to N removal. The response of N removal processes to changing environmental conditions may, at least in the short term, depend on the properties of the established microbial communities. Therefore, these responses are expected to differ between lake sediments with contrasting N cycling microbial networks.

We suggest two hypotheses for further study: that the *nirS*:*nirK* ratio should be considered a possible indicator of N removal efficiency and that oligotrophic lake sediments harbor higher microbial diversity. Finally, we report the first evidence of possible comammox *Nitrospira* in oligotrophic Lake Sediments in Switzerland.

This study not only provides a comprehensive view on the biology and ecology that underpins N removal in freshwater lakes, which is an important ecosystem service, but also shows the value of including metagenomic analysis to better understand the microbially mediated N cycle in natural systems.

## MATERIALS AND METHODS

### Study sites.

Lakes Baldegg and Sarnen are situated on the Swiss plateau and in the peri-alpine region, respectively. Both are small lakes, with a surface of 7.15 km^2^ and a maximum depth of 49 m for Lake Sarnen and 5.22 km^2^ and 66 m for Lake Baldegg (20). Lake Sarnen is surrounded by mountains, forests, and extensive agriculture and therefore remained oligotrophic throughout the 20th century, maintaining a total phosphorus (TP) concentration of ∼5 mg P m^−3^. Lake Baldegg, in comparison, receives high P loads due to pig and cattle farming in the catchment. In the second half of the 20th century, this lake experienced pronounced eutrophication that peaked in the 1970s. To prevent bottom water anoxia, the lake has been artificially aerated with O_2_ during the stratified season since 1982 (71). Today, the lake is still eutrophic with TP concentrations of ∼24 mg P m^−3^ (37, 72). Sediment trap data from our parallel study in 2017 and 2018 found a higher sedimentation rate of N-enriched OM in Lake Baldegg (56.8 g C m^−2 ^year^−1^ and 11.2 gN m^−2 ^year^−1^, C:N 5.9) compared to Lake Sarnen (28.6 g C m^−2 ^year^−1^ and 4.1 gN m^−2 ^year^−1^, C:N 8.5) (20). Both lakes are dimictic with an oxic water column throughout the year; thus, the oxic-anoxic transition zone, where most N transformation processes occur, extends over the first few millimeters of the sediments. To study spatial variability, we selected five sampling locations representing different depths between the inflow and outflow of the lakes (Fig. 1). The seasonal dynamics were investigated by sampling each station in March, May, August, and September 2018 (for details, see Müller et al. [20]). The inflow location of Lake Baldegg was additionally sampled in August and September. Sediment depth was resolved by analyzing pore water through sampling ports or cutting layers from the core, as described below.

### Sediment sampling and chemical and statistical analyses.

The sediment sampling, pore water analyses, and oxygen measurements are described in Müller et al. (20). Briefly, we used a gravity corer for the sediment sampling with PVC tubes (5.9-cm diameter and 60-cm length; Uwitec, Austria). The PVC tubes had predrilled ports to extract pore water samples (∼200 μl) in a 0.25-cm depth resolution using MicroRhizon filter tubes (0.2 μm pore size; 0.8 mm diameter; Rhizosphere Research Products, Wageningen, Netherlands). The pore water was sampled onshore immediately after core retrieval, transferred to 1.5-ml tubes, stored on ice in the dark, and analyzed within 24 h. NO_3_^−^, NO_2_^−^, SO_4_^2−^, and NH_4_^+^ were analyzed using two ion chromatography devices (cations, 882 Compact IC plus; anions, 881 Compact IC pro; Metrohm, Switzerland). In addition, vertical O_2_ concentration profiles were recorded with an O_2_ micro-optode mounted to an automated micromanipulator (Presens, Germany) immediately after core retrieval. The O_2_ and pore water profiles were visualized using ggplot2, and the spatial and temporal differences were calculated using analysis of variance (ANOVA) from the base statistical packages in R (version 3.6.1) (73, 74).

### DNA sampling and extraction.

During each sampling event, a sediment core was retrieved for DNA analysis. The core was cut with a metal slicing device into the following sections: 0 to 0.5 cm, 0.5 to 1 cm, 1 to 1.5 cm, 1.5 to 2 cm, 2 to 3 cm, and 3 to 5 cm. The slicing device was washed with 80% ethanol and molecular grade water (DNase/RNase free; SigmaAldrich) before each core sampling and with molecular-grade water during the slicing of cores. Three subsamples (∼2 ml) were taken from each section with a 3-ml syringe and immediately fixed in 2 ml of RNAlater (Sigma Life Science) in a 5-ml tube (DNase/RNase free; Qiagen). The sediment samples were kept at 4°C until the next day and then stored at −80°C. Prior to nucleic acid extraction, samples were thawed and washed three times with 2.5 ml 1× Tris-EDTA buffer to remove excess salt from RNAlater. Nucleic acids were then extracted using the RNeasy PowerSoil total RNA kit in combination with the RNAeasy PowerSoil DNA elution kit (Qiagen) according to the manufacturer's instructions. The DNA quality and quantity were measured with a NanoDrop spectrophotometer (NanoDrop One), and extracts were stored at −80°C until sequencing. Extraction blanks were prepared to check for contamination and used as negative controls in sequencing.

### Amplicon sequencing, pipeline, and statistical analysis.

All amplicon sequencing was performed by Novogene (Hong Kong) by following standard sequencing and quality control protocols. Extracted DNA from all stations and all seasons (120 samples from Lake Baldegg and 96 samples from Lake Sarnen) was amplified with universal 16S rRNA gene primers 341F (5′-CCT AYG GGR BGC ASC AG-3′) and 806R (5′-GGA CTA CNN GGG TAT CTA AT-3′), targeting the V3-V4 region (fragment length, ∼466 bp), and sequenced with Illumina HiSeq technology to generate paired-end reads. This resulted in 119 samples with an average of 112,857 raw paired-end reads in Lake Baldegg and 90 samples with an average of 139,822 raw paired-end reads in Lake Sarnen. The sequences were analyzed separately for each lake using the DADA2 pipeline in R (version 3.5.1) (74, 75). Briefly, after a quality check, the raw sequence adapters were trimmed and dereplicated, and error rates were calculated before applying the DADA2 core sample inference algorithm. Next, the forward and reverse reads were merged to obtain the amplicon sequence variants (ASVs). Following chimera removal with the removeBimeraDenovo algorithm, the taxonomic assignment of the ASVs was performed using a Naïve Bayesian classifier method based on the SILVA database 132 for the V3-V4 region (76). (Note that the SILVA database release 132 implemented numerous changes to the taxonomy and, e.g., assigned the former *Betaproteobacteria* to the class of *Gammaproteobacteria*). Unclassified and rare ASVs (less than five instances in at least 10% of all samples) were filtered out. The ASV abundance tables and the corresponding environmental parameters were further analyzed by calculating the alpha and beta diversity (redundancy analysis [RDA]), testing for significantly different microbial community composition between the lakes, locations, sampling months, and sediment depth using adonis and visualized with the phyloseq, ggplot2, vegan, and microbiomeSeq packages using R statistical software (version 3.6.3) (73, 74, 77–79). Details of the 16S rRNA gene amplicon library can be found in the supplemental material (Table S1), and the abundance table was added to the Eawag data package (see “Data availability,” below).

10.1128/msphere.01013-21.5 TABLE S1 Sequencing overview for the 16S amplicon sequencing data (PE, paired end). Download Table S1, XLSX file, 0.01 MB. Copyright © 2022 Baumann et al. 2022 Baumann et al. https://creativecommons.org/licenses/by/4.0/ This content is distributed under the terms of the Creative Commons Attribution 4.0 International license.

### Metagenomic sequencing, pipeline, and statistical analysis.

For metagenomic sequencing, DNA extracts from all samples of one core were pooled using 1 μg of DNA from each sampling depth (0 to 0.5 cm, 0.5 to 1 cm, 1 to 1.5 cm, 1.5 to 2 cm, and 2 to 3 cm) after measuring the DNA concentration with a Qubit (Qubit 2.0 fluorometer; Invitrogen). We obtained eight metagenomes from Lake Sarnen (deepest and shallow_inflow station for March, May, August, and September) and 10 metagenomes from Lake Baldegg (deepest and shallow_outflow locations for March, May, August, and September and shallow_inflow for August and September). The metagenomes were sequenced using the Illumina NextSeq platform to generate 150-bp paired-end reads (averaging ∼350 bp in length) by Novogene (Hong Kong). The quality of each metagenome was evaluated using FastQC (80). Prinseq was used to trim metagenomic reads (minimum quality mean, 20) (81). Each sample was assembled using Megahit with the options meta-sensitive and min_contig length 500 (82), and reads were mapped back using BBmap (minid = 0.95) (83). The sam files were converted to bam files using sambamba (84) and SAMtools (85), and the reads were subsequently counted using featureCounts from subread 1.6.4 (86). For functional annotation, we used prokka v1.13 (87). The N transformation genes (Table 2) were obtained by mapping the protein output from prokka against the N cycle database (NCycDB; accessed July 2019), applying a 100% identity criterion (32). The genes and representative gene families were assigned to N cycle processes based on information provided by NCycDB (see Table 1 in Tue et al. [32]); if a gene was assigned to more than one process in this table, we made a new category naming all processes (e.g., Denitrification_DNRA) (Table 2). Further analysis and visualization were done in R (version 3.6.3) (74). The quantified genes were normalized for gene lengths and sequencing depth by calculating genes per million (gpm) for the visualization. We calculated the *nirS*:*nirK* ratio from the gpm data to indicate the ratio of complete denitrifiers versus incomplete denitrifiers (29). A principal component analysis was used to test for significant differences in the abundance of N transformation genes between lakes (alpha = 0.001) using DESeq2 (88). We used the nonnormalized feature count files because DESeq2 applies its own normalization step (by following the workflow from Love et al., accessed 1 June 2019 [89]). We used the Wald test, with the condition Lake Baldegg versus Lake Sarnen, to extract the significantly different N transformation genes and the Benjamini-Hochberg method from DESeq2 to correct *P* values for multiple testing (88, 89). The results were visualized with ggplot2 and ComplexHeatmap (90) after calculating the *z* score [*z* = (*x* − mean(*x*))/SD(*x*), where *x* is N gene counts and SD is standard deviation] for all significantly different N transformation genes to emphasize the differences. Finally, candidate key players containing the significantly different genes and *nirS* were resolved by taxonomic assignment of the prokka output using Kaiju (91). Kaiju is a taxonomic assignment tool developed for short reads and might be biased when used for long reads such as assembled contigs. Kaiju still contains the former class *Betaproteobacteria*; we assigned it manually to *Gammaproteobacteria*, considering the taxonomical assignment of the SILVA database 132.

**TABLE 2:** Overview of the nitrogen transformation pathways, processes, and corresponding gene families analyzed using NCycDB^*a*^

Pathway	Enzyme	Gene family
Nitrification	Ammonia monooxygenase	*amoABC*
	Hydroxylamine dehydrogenase	*hao*
	Nitrite oxidoreductase	*nxrAB*
Denitrification or DNRA	Nitrate reductase	*napABC*, *narGHJI*, *narZYVW*
DNRA	Nitrite reductase	*nirBD*, *nrfABCD*
Denitrification	Nitrite reductase	*nirK* (Cu), *nirS* (cytochrome *cd*_1_)
	Nitric oxide reductase	*norBC*
	Nitrous oxide reductase	*nosZ*
Anammox	Hydrazine oxidoreductase	*hzo*
	Hydrazine synthase	*hzsABC*
	Hydrazine dehydrogenase	*hdh*
ANRA	Nitrate reductase	*nasAB*, *NR*, *narBC*
	Nitrite reductase	*nirA*
Nitrogen fixation	Nitrogenase	*nifDHKW*, *anfG*
Organic nitrogen degradation	Urease	*ureABC*
	Nitroalkane oxidase	*nao*
	Nitronate monooxygenase	*nmo*
	Glutamate dehydrogenase	*gdh*
	Glutamate synthase	*gs*
	Glutaminase	*glsA*
	Glutamine synthetase	*glnA*
	Asparagine synthase	*asnB*
	Glutamin-(aspargin-)ase	*ansB*
Others	Hydroxylamine reductase	*hcp*
	Particulate methane monooxygenase	*pmoABC*

a Data are adapted from Tu et al. (32). Categories of pathways are exclusive if not stated otherwise.

The sequencing depth, quality, filtered reads, mean contig length, and mapped reads for each sample are summarized in the supplemental material (Table S2).

### Data availability.

All sequences can be found under the NCBI BioProject PRJNA726540. The 119 16S rRNA gene sequences and the metadata from Lake Baldegg have the accession numbers SAMN18962412–SAMN18962530. The 90 16S rRNA gene sequences and the metadata of Lake Sarnen are deposited under the accession numbers SAMN18978354–SAMN18978443. The 10 metagenomes and the metadata can be found under the accession numbers SAMN19067306–SAMN19067315. The eight metagenomes and the metadata from Lake Sarnen can be found under the accession numbers SAMN19030157–SAMN19030164. All other data, such as metadata and the abundance tables, are available at the institutional data repository of Eawag (ERIC) (https://opendata.eawag.ch/) in accordance with FAIR data sharing principles (https://doi.org/10.25678/0005HX).
