# Supplementary figures and images for: Microbial Nitrogen Transformation Potential in Sediments of Two Contrasting Lakes Is Spatially Structured but Seasonally Stable
Source: mSphere. 2022 Feb 2;7(1):e01013-21. doi: 10.1128/msphere.01013-21 (PMC8809388; doi:10.1128/msphere.01013-21)

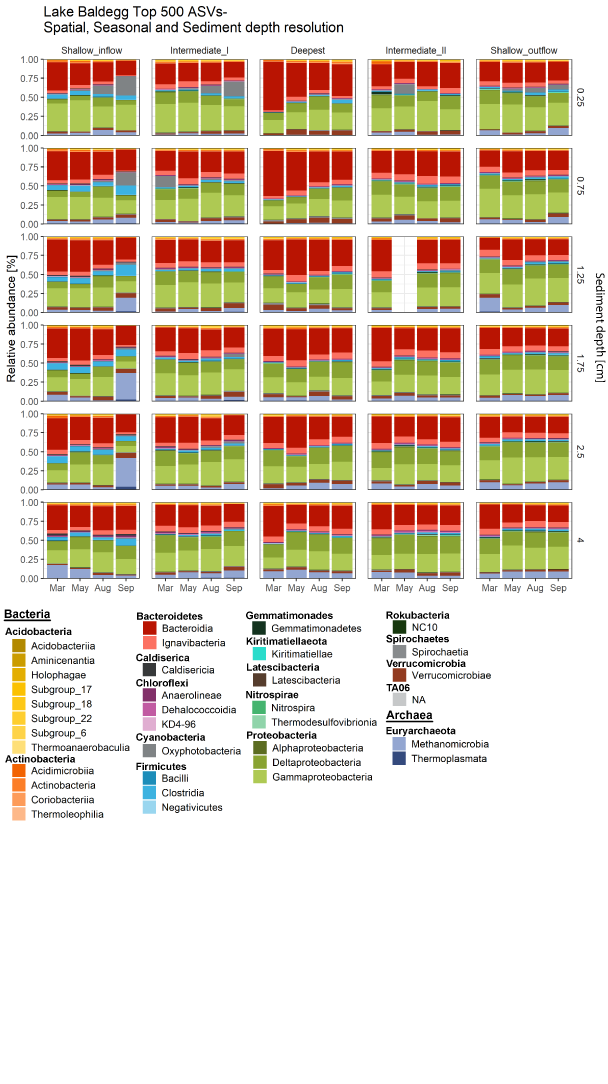

Supplement: FIG S3 [file msphere.01013-21-sf003.tif]

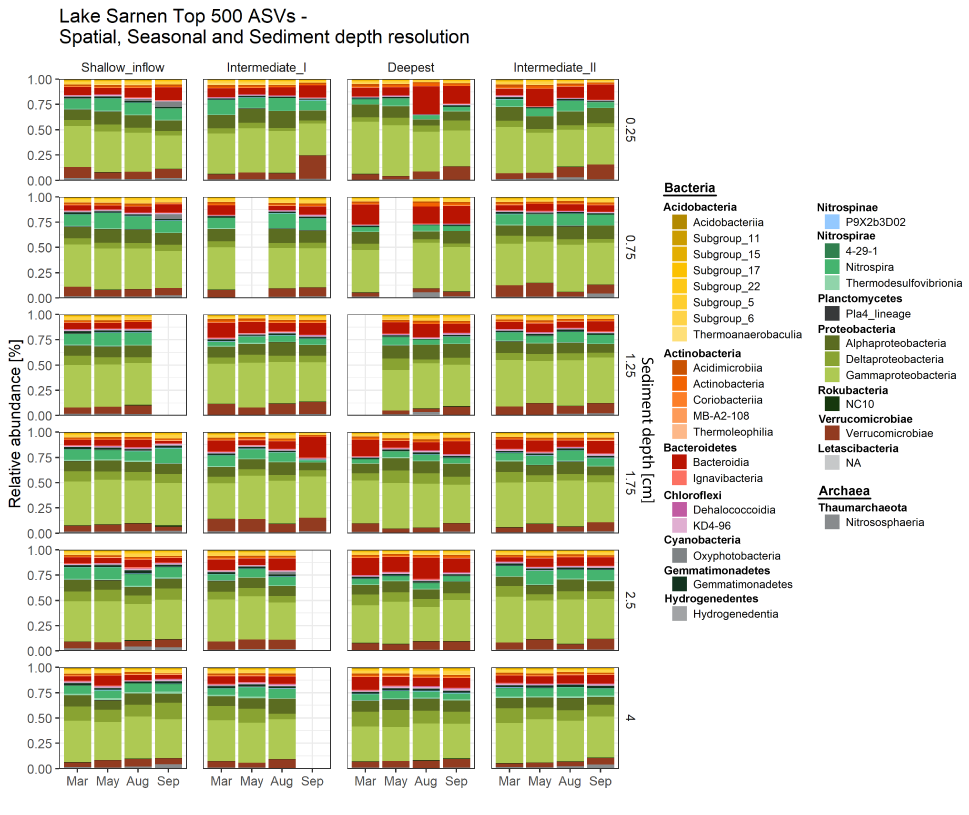

Supplement: FIG S4 [file msphere.01013-21-sf004.tif]
